# Sentinel headache as a warning symptom of ischemic stroke

**DOI:** 10.1186/s10194-020-01140-3

**Published:** 2020-06-10

**Authors:** Elena R. Lebedeva, Anton V. Ushenin, Natalia M. Gurary, Denis V. Gilev, Jes Olesen

**Affiliations:** 1 Department of Emergency Neurology, the Ural State Medical University, Repina 3, Yekaterinburg, 620028 Russia; 2 International Headache Center “Europe-Asia”, Yekaterinburg, Russia; 3 Medical Union “New Hospital”, Yekaterinburg, Russia; 4 Department of Econometrics and Statistics, the Graduate school of Economics and Management, the Ural Federal University, Yekaterinburg, Russia; 5 Danish Headache Center, Department of Neurology, Rigshospitalet-Glostrup, University of Copenhagen, Copenhagen, Denmark

**Keywords:** Stroke, Migraine, Headache, Cerebrovascular disease, Sentinel headache

## Abstract

**Background:**

There are no previous controlled studies of sentinel headache in ischemic stroke. The purpose of the present study was to evaluate the presence of such headache, its characteristics and possible risk factors as compared to a simultaneous control group.

**Methods:**

Eligible patients (*n* = 550) had first-ever acute ischemic stroke with presence of new infarction on *magnetic resonance* imaging with diffusion-weighted imaging (*n* = 469) or on computed tomography (*n* = 81). As a control group we studied in parallel patients (*n* = 192) who were admitted to the emergency room without acute neurological deficits or serious neurological or somatic disorders. Consecutive patients with stroke and a simultaneous control group were extensively interviewed soon after admission using validated neurologist conducted semi-structured interview forms. Based on our previous study of sentinel headache in transient ischemic attacks we defined sentinel headache as a new type of headache or a previous kind of headache with altered characteristics (severe intensity, increased frequency, absence of effect of drugs) within seven days before stroke.

**Results:**

Among 550 patients with stroke 94 patients (17.1%) had headache during seven days before stroke and 12 (6.2%) controls (*p* < 0.001; OR 3.9; 95% CI 1.7–5.8). Totally 81 patients (14.7%) had sentinel headache within the last week before stroke and one control. Attacks of arrythmia during seven days before stroke were significantly associated with sentinel headache (*p* = 0.04, OR 2.3; 95% CI 1.1–4.8).

**Conclusions:**

A new type of headache and a previous kind of headache with altered characteristics during one week before stroke are significantly more prevalent than in controls. These headaches represent sentinel headaches. Sudden onset of such headaches should alarm about stroke.

## Introduction

Sentinel headache before a subarachnoid haemorrhage (SAH) is well described as a sudden, intense, persistent headache, with features different from any usual previous headache. It precedes subarachnoid haemorrhage by days or weeks and occurs in 15–60% of patients with spontaneous SAH [1]. Not only patients with SAH may, however, have sentinel headache. Sentinel headache means a headache that warns against an impending disease. In our previous study it was found in transient ischemic attacks (TIA) [2]. We defined headache as a new type of headache that had never been experienced before or as a previous headache with changes of characteristics occurring within 7 days of TIA. Such headaches were experienced by 22 out of 120 (18,3%) patients with TIA [2].

There are no previous studies of sentinel headache in ischemic stroke within the last 30 years to the best of our knowledge. But before that Gorelick P.B. and coworkers described the phenomenon in an uncontrolled study of 150 patients with ischemic stroke using stroke registry records but not direct professional interview [3]. The present report is part of a broader study on headache in ischemic stroke compared to a simultaneous control group using semi-structured face-to-face interview by a neurologist. The focus of this report is on headache occurring within 7 days of ischemic stroke. Based on our previous experience with TIA we subdivided headaches into those previously encountered with- or without change in characteristics and new headache never before encountered. We compared patients with sentinel headache to patients without sentinel headache, evaluated triggers and probable risk factors and finally gave clinical recommendations enabling the practicing neurologist to identify patients with sentinel headache.

## Material and methods

The results presented here derive from the large Yekaterinburg study of headache in cerebrovascular disorders.

### Study population and design

This prospective study was conducted from September 2012 to October 2015. The clinical part of the study was performed at city hospital “New Hospital”, Yekaterinburg, Russia, using questionnaires and procedures developed by Elena R. Lebedeva and Jes Olesen.

Eligible patients had ischemic stroke with presence of new infarction on *magnetic resonance* imaging (MRI) with diffusion-weighted imaging (DWI) or on computed tomography (CT). Most of them (75%) were admitted to the emergency department 12–24 h after the onset of symptoms of stroke, several within 3 h (15%) and others (10%) 4–12 h after stroke. The criteria for inclusion in the study were as follows: ischemic stroke on CT or DWI evidence of relevant infarction; no history of any previous stroke or transient ischemic attack, no other serious pathology of the nervous system (brain tumor, traumatic brain injury, multiple sclerosis, epilepsy, encephalitis, meningitis, etc.) and no serious somatic pathology; no impaired consciousness and no memory or speech impairments that impeded the collection of information. The participants could give a clear description of their headaches and accepted follow-up for five years for the study of poststroke persistent headache and cardiovascular events after stroke. Excluded were: patients comatose/stuperose/intubated after admission, patients not consenting for examinations, patients with dementia, cognitive impairment (Mini-Mental State Examination less than 26) and aphasia, patients not able to identify time of onset of headache and stroke symptoms.

As a control group we studied in parallel patients matched by age to patients with stroke who were admitted to the emergency room without acute neurological deficits or serious neurological or somatic disorders.

### Evaluation

We used the “gold standard” for headache diagnosis — a semi-structured face-to-face interview by a neurologist. An interview with one patient took 90 min. Two neurologists collected patient data prospectively, using a standardized case-report form as soon as the necessary clinical examination and MRI or CT were completed. Sociodemographic characteristics of patients, medical history, date and time of stroke and headache onset, characteristics of stroke (clinical symptoms, arterial territory, National Institutes of Health Stroke Scale (NIHSS) score at initial presentation, etiology), results of neurological examination. Headache during seven days before stroke and past history of headache (migraine with and without aura, tension type headache, cluster headache, medication overuse headache, etc.) were recorded using extensive semi-structured interview forms. We asked about the presence and the exact day of development of any headache during seven days before stroke, and about presence of any headache during one year before stroke (excluding one week before stroke). We asked patients to describe characteristics of their headaches: location, side, frequency, the presence of headache which reached its most intense pain within 1 min, duration of headache, character (pressing, pulsation, dull, etc.), severity (mild, moderate, severe), aggravation by physical activity, presence of aura, and its characteristics, accompanying symptoms, effect of drugs for pain relief, the name of drugs and frequency of their use, etc. If headaches arose on the day of stroke, we asked about the time and compared with the time of stroke. The same interview about previous headache during one year (excluding the last week) and headaches during seven days before admission to the hospital was taken in controls. During this interview we also asked about age of any headache onset. After the interview we compared characteristics of previous headache during one year with headache during seven days before stroke and at admission and made a judgment about change of headache characteristics or development of a new type of headache.

Results of consultations of specialists (cardiologist, endocrinologist, vascular surgeon, etc.), imaging, other investigations and laboratory tests were also recorded in the case-report form as well as previous and current treatment.

Besides we evaluated the presence of the following possible trigger factors for stroke and headache during seven days before stroke which were chosen using our previous studies and available literature [4]: acute alcohol abuse (alcohol intake of more than 40 g (equivalent to 4 standard drinks) or more than 150 g (equivalent to 15 standard drinks), heavy physical exertion (lifting heavy weights, sportive competitions, unusual active physical exercise, etc.), psychological stress (stressful life events and negative affect), overwork, lack of sleep, hypertensive crisis (systolic blood pressure of 180 mm Hg (mm Hg) or higher and diastolic blood pressure of 120 mmHg or higher), clinical infections (diagnosis was based on the presence of fever alone, typical symptoms alone, or fever with typical symptoms and general practitioner consultation), attack of arrhythmia (patient felt and described severe unusual palpitations or feeling a pause between heartbeats, heart rate above 100 beats per minute or below 60 per minute, *presence of arrythmia on electrocardiogram*), overheating (bath, sauna, etc.). We also investigated the presence during the last year the following risk factors which can be associated with stroke and headache: female sex, smoking (current smoker), consumption of light alcoholic beverages (at least 0.5 l per week) and strong alcoholic beverages (150 g per week), increased body mass index (BMI > 25), low physical activity (less than 30 min of physical exercises 1 time per week), family history of stroke (one or more first degree relatives with stroke) and presence of disorders which had been diagnosed in patients before stroke: arterial hypertension, diabetes mellitus, atrial fibrillation, angina pectoris, myocardial infarction, peripheral artery disease, migraine, tension type headache, large-artery atherosclerosis (≥ 50% stenosis or occlusion of arteries on the neck which were verified during triplex ultrasonography or CT-angiography).

### Definitions

The diagnoses of previous and present headaches were made according to the explicit diagnostic criteria of the International Headache Society, the International Classification of Headache Disorders (ICHD)-3 [5]. We recorded headache within the last year and within the last week before stroke and also in controls within the last year and the last week before admission to the hospital. We distinguished between previous headache without change of characteristics, headache with altered characteristics and a new type of headache. We defined a new type of headache as a headache which arose for the first time in the week before stroke. Migraine or tension type headache with changes of characteristics within the last week before stroke as well as migraine or tension type headache as a new type of headache were defined as migraine-like headache and tension- type-like headache because they were considered secondary to stroke. Based on our previous study of TIA we defined sentinel headache as a new type of headache or a previous kind of headache with altered characteristics (severe intensity, increased frequency, absence of effect of drugs) which arose within seven days before stroke.

### Statistical analysis

Statistical analyses were performed with Stata (ver.14.0) and Microsoft Excel (2014). The basic comparison was between patients with ischemic stroke and controls. Some comparisons were done between patients with and without sentinel headache. Crude prevalence of headache disorders was calculated in percentages, odds ratio (OR). Continuous variables were summarized as means, and categorical variables as numbers and percentages. We used chi-squared to compare distributions of categorical variables between groups. When quantitative indicators were evaluated for compliance with a normal distribution, we used the Shapiro-Wilk test (when the number of studied was less than 50) or the Kolmogorov-Smirnov test (when the number of investigated was more than 50). In the case of analysis of Chi-square calculation, if the expected phenomenon in at least one cell was less than 10, we calculated the χ2 criterion with Yates’s correction, which reduces the probability of error of the first type, i.e. detecting differences where there are none. We set statistical significance at *P* < 0.05. Univariable analyses were performed to calculate crude odds ratios with 95% confidence intervals (CI). The differences between prevalence of trigger and probable risk factors between patients with and without sentinel headache were statistically examined by an unpaired t-test and chi-square test. A 2-tailed P-value < 0.05 was statistically significant. We could not calculate the necessary sample size since nothing was known about sentinel headache in ischemic stroke. As it turned out our large material was enough for highly significant results. All analyses were performed by a statistician (DVG).

## Results

### Characteristics of patients with ischemic stroke and controls

A total of 2995 participants with ischemic stroke were prospectively examined and 2445 patients were excluded: 933 (38.1%) patients had previous cerebrovascular accident, 541 (22.1%) had memory problems, 415 (16.9%) had impaired consciousness, 265 (10.8%) had aphasia, 202 (8.2%) had hemorrhagic stroke, in 89 (3.6%) of patients MRI of the brain did not reveal an ischemic lesion. Thus, the study included 550 participants (306 men and 244 women) aged 25 to 89 years, mean age 63,1 (±11,4). 469 had MRI with DWI and 81 had CT. We examined also 225 controls. 33 patients were excluded, and 192 patients were included.

Table 1 shows clinical characteristics of 550 patients with stroke compare to controls (*n* = 192). The mean age of patients with stroke and controls was without significant difference (63.1 and 58.7 respectively), age range of patients of both groups was almost the same (25–89 in stroke patients and 26–87 in controls). The following factors were significantly associated with stroke: male sex, smoking, intake of strong alcohol beverage, arterial hypertension, diabetes mellitus, hyperglycemia, atrial fibrillation, low physical activity, hypercholesterinemia, angina pectoris, myocardial infarction and family history of stroke (Table 1).

**Table 1:** Clinical characteristics of patients with stroke compare to controls

Characteristics	Patients with stroke (*n* = 550)	Controls (*n* = 192)	Р,OR, 95% CI
Mean age	63.1	58.7	0.1
Males, n (%)	298 (54.2%)	69 (35.9%)	< 0.001; 2.1; 1.5–3.0
Smokers, n (%)	236 (42.9%)	45 (23.4%)	< 0.001; 2.5; 1.7–3.6
Low alcohol beverage, n (%)	45 (8.2%)	11 (5.7%)	0.3
Strong alcohol beverage, n (%)	118 (21.5%)	18 (9.4%)	< 0.001; 1.5; 0.7–2.9
Arterial hypertension, n (%)	514 (93.5%)	108 (56.3%)	< 0.001; 7.1–17.3
Diabetes mellitus, n (%)	84 (15.3%)	14 (7.3%)	0.007; 2.3; 1.3–4.1
Hyperglycemia, n (%)	206 (37.5%)	26 (13.5%)	< 0.001; 3.8; 2.4–6.0
Atrial fibrillation, n (%)	85 (15.5%)	7 (3.6%)	< 0.001; 4.8; 2.2–10.6
Body mass index > 25, n (%)	373 (67.8%)	118 (61.5%)	0.1
Low physical activity, n (%)	207 (37.6%)	28 (14.6%)	< 0.001; 3.5; 2.3–5.5
Stroke in first degree relatives n (%)	207 (37.6%)	48 (15.0%)	0.002; 1.8; 1.3–2.6
Peripheral artery disease, n (%)	13 (2.3%)	1 (0.5%)	0.2
Hypercholesterinemia, n (%)	230 (41.8%)	55 (28.6%)	0.002; 1.8; 1.3–2.6
Angina pectoris, n (%)	149 (27.1%)	18 (9.4%)	< 0.001; 3.6; 2.1–6.0
Myocardial infarction, n (%)	53 (9.6%)	7 (3.6%)	0.01; 2.8; 1.2–6.3

Almost half of the participants in both groups were pensioners and the other half were employed. All patients with stroke lived in Yekaterinburg as well as 97% controls. Most patients in both groups had middle social status. The biggest number of patients (82.9%) had stroke in the anterior circulation and 17.1% of patients in posterior circulation. Distribution of patients with stroke according to Trial of Org 10,172 in Acute Stroke Treatment (TOAST) classification and according to National Institutes of Health Stroke Scale (NIHSS) score is presented in Table 2: 76.5% of patients had NIHSS score less than 8 and most patients (32.4%) had stroke of undetermined etiology.

**Table 2:** Distribution of patients with stroke according to TOAST classification and NIHSS score

Distribution of stroke	Males (*n* = 298)	Females (*n* = 252)	All (*n* = 550)
**TOAST classification**			
Large-artery atherosclerosis	97 (32.5%)	57 (22.6%)	154 (28.0%)
Small-vessel occlusion (lacune)	60 (20.1%)	61 (24.2%)	121 (22.0%)
Cardioembolism	37 (12.4%)	48 (19.0%)	85 (15.5%)
Stroke of undetermined etiology	96 (32.3%)	82 (32.6%)	178 (32.4%)
Other etiologies	8 (2.7%)	4 (1.6%)	12 (2.1%)
**NIHSS score**			
Less than 8 points	223 (78.4%)	198 (78.6%)	421 (76.5%)
8–16 points	74 (24.8%)	53 (21.0%)	127 (23.1%)
More than 16 points	1 (0.3%)	0 (0%)	1 (0.2%)

Control patients were admitted to the emergency room with the following diagnoses: “lumbago” or “lumbar spine osteochondrosis” (*n* = 99), “pancreatitis “(*n*= 62), “gastrointestinal ulcer” (*n* = 7), tick bite (*n* = 14), irritable bowel syndrome (*n* = 2), paroxysmal benign positional vertigo (*n* = 2), arthritis (*n* = 5), allergic reaction (*n* = 1).

### Headache within the last year (excepting the last week) before ischemic stroke

The prevalence of all primary headache disorders in patients with stroke during the last year before stroke (excepting the last week) and in controls is presented in Table 3. A past history of migraine without aura was seen in 13.3% of patients with ischemic stroke and in 8.9% of controls. Past history of TTH was found in 65.2% of patients with stroke and 70.3% of control patients. Only the prevalence of migraine in females with stroke during the last year was significantly higher than in controls: 23.0% and 13.0% respectively (*p* = 0.03, OR 2.0, 95% CI 1.1–3.7).

**Table 3:** The prevalence of headache disorders in patients (*n* = 550) during the year before stroke (excepting last week before stroke) and during the last year before interview in controls (excepting last week before interview) (*n* = 192) according to ICHD-3

Type of headache	Males with stroke (*n* = 298)	Male controls (*n* = 69)	Р, OR (95% CI)	Females with stroke (*n* = 252)	Female controls (*n* = 123)	Р, OR (95% CI)	All patients with stroke (*n* = 550)	All controls (*n* = 192)	Р, ОR, 95% CI
Migraine without aura	9 (3.0%)	1 (1.4%)	0.8	37 (14.7%)	14 (11.4%)	0.5	46 (8.4%)	15 (7.8%)	0.8
Migraine with aura	3 (1.0%)	0	0.9	5 (2.0%)	1 (0.8%)	0.7	8 (1.5%)	1 (0.52%)	0.5
Chronic migraine	3 (1.0%)	0	0.9	16 (6.3%)	1 (0.8%)	**0.03** **8.3; 1.1–63.1**	19 (3.5%)	1 (0.52%)	0.06
**All migraine**	15 (5.3%)	1 (1.4%)	0.3	58 (23.0%)	16 (13.0%)	**0.03** **2.0; 1.1–3.7**	73 (13.3%)	17 (8.9%)	0.1
Episodic TTH***	151 (50.7%)	48 (69.6%)	**0.007** **0.5; 0.3–0.8**	173 (68.7%)	84 (68.3%)	0.9	324 (58.9%)	132 (68.7%)	**0.02** **0.7; 0.5–0.9**
Chronic TTH	8 (2.7%)	0 (0%)	0.4	12 (4.8%)	3 (2.4%)	0.4	20 (3.6%)	3 (1.6%)	0.2
All TTH	159 (53.4%)	48 (69.6%)	**0.02** **0.5; 0.3–0.9**	185 (73.4%)	87 (70.7%)	0.7	344 (62.5%)	135 (70.3%)	0.06
Cluster headache	2 (0.7%)	0 (0%)	0.8	0 (0%)	1 (0.81%)	0.7	2 (0.4%)	1 (0.52%)	0.7
Medication overuse headache	1 (0.3%)	0 (0%)	0.4	6 (2.4%)	0 (0%)	0.2	7 (1.3%)	0 (0%)	0.3
No headache	132 (44.3%)	24 (34.7%)	0.2	48 (19.0%)	15 (12.2%)	0.1	180 (32.7%)	39 (20%)	**0.002** **1.9; 1.3–2,8**

### Headache within the last week before ischemic stroke

Among 550 patients with stroke 94 patients (17.1%) had headache during seven days before stroke and 12 (6.2%) of controls (*p* < 0.001; OR 3.9; 95% CI 1.7–5.8). Types of headache during 7 days before stroke compared to controls is presented in Table 4. We compared their characteristics with previous headache and subdivided them in three groups: 1) headaches without changes of characteristics; 2) headache with altered characteristics (change of character, frequency, duration, severity and accompanying symptoms); 3) new type of headache which arose for the first time. According to these data 13 patients (2.4%) with stroke and 10 control patients (5.2%) had *previous headaches without changes of characteristics*, the difference was not significant (*p* = 0.09). *Headache with altered characteristics* had 54 (9.8%) with stroke and two (1%) control patients (*p* < 0.001). Among these headaches with changes of characteristics only the prevalence of *tension type-like headache* had significant difference between two groups: 41 (7.5%) patients with stroke and two (1.0%) control patients (*p* < 0.001). These headaches became severe, longer lasting, more frequent and sometimes patients had new accompanying symptoms. In some patients analgesics became ineffective for pain relief. A *New type of headache* during seven days before stroke had 27 stroke patients (4.9%) and no controls (*p* = 0.004). *Migraine-like headache* was seen in 20 (3.6%) patients before stroke and in no controls (*p* = 0.02). Two of the new headaches (0.4%) were *thunderclap headaches* which were not seen in controls. This headache arose one day before stroke, reached maximum in one minute and lasted 60 min, was bilateral, very severe, localized in all the head in one patient and in the occipital region in other. All drugs for pain relief were ineffective. A new type of headache and a previous headache with altered characteristics during seven days before stroke are thus much more prevalent in stroke patients than in controls and therefore causally related to stroke. These headaches represent sentinel headache.

**Table 4:** Types of headaches during seven days before stroke and during seven days before interview in controls

Type of headache	Previous headache without changes of characteristics in patients with stroke (*n* = 550)	Previous headache without changes of characteristics in controls (*n* = 192)	Р, OR, 95% CI	Previous headache with altered characteristics in patients with stroke (*n* = 550)	Previous headache with altered characteristics in controls (*n* = 192)	Р, OR, 95% CI	New type of headache in patient with stroke (*n* = 550)	New type of headache in controls (*n* = 192)	P, OR, 95% CI
Migraine without aura	1 (0.2%)	0 (0%)	0.6	12 (2.2%)	0 (0%)	0.08	20 (3.6%)	0 (0%)	**0.02**
Migraine with aura	0 (0%)	0 (0%)	–	1 (0.2%)	0 (0%)	0.6	0 (0%)	0 (0%)	
All migraine	1 (0.2%)	0 (0%)	0.6	13 (2.4%)	0 (0%)	0.07	20 (3.6%)	0 (0%)	**0.02**
TTH^a^	12 (2.2%)	10 (5.2%)	0.06	41 (7.5%)	2 (1.0%)	**< 0.001 8.9; 2.1–36.9**	5 (0.9%)	0 (0%)	0.4
Cluster headache	0 (0%)	0 (0%)	–	0 (0%)	0 (0%)	–	0 (0%)	0 (0%)	
Thunderclap headache	N/A^b^	N/A		N/A	N/A		2 (0.4%)	0	0.9
All headaches	13 (2.4%)	10 (5.2%)	0.09	54 (9.8%)	2 (1.0%)	**< 0.001 10.3; 2.5–42.8**	27 (4.9%)	0 (0%)	**0.004**

^a^Migraine with and without aura with altered characteristics and tension headache with altered characteristics, as well as migraines with and without aura and tension headache in the form of a new type of headache, were defined in the text as a migraine-like and tension type-like headache, respectively, since they can be attributed to stroke

^b^*N/A* not acceptable

### Sentinel headache as a warning of ischemic stroke

**Fig. 1:**
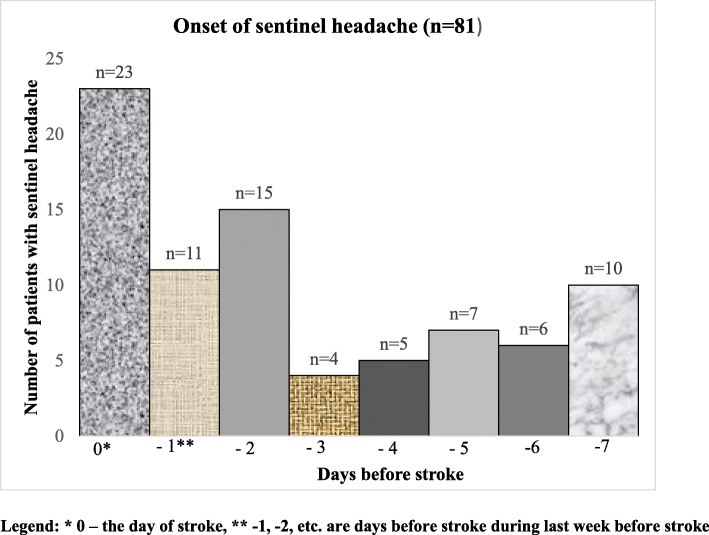
Onset of sentinel headache during 7 days before ischemic stroke

Among 550 patients totally 81 (14.7%) had *sentinel headache* within the last week before stroke. This group included 52 females and 29 males. Their mean age was 62.4 years (66.0 in females and 55.9 in males). 70 patients had stroke in anterior circulation and 11 in posterior circulation. Among 81 patients with sentinel headache 46 had tension type-like headache, 33 migraine-like headache and two thunderclap headache. Headache arose from 60 min to 10 h before stroke in 23 patients (28.4%), 1–2 days before stroke (26 patients, 32.1%), and all others had headache during 3–7 days before stroke (Fig. 1). In most patients (56 patients, 69.1%) headaches disappeared within 24 h of onset, in 16 patients (19.7%) they continued up to 48 h, in seven patients (8.6%) up to four days, in two patients (2.5%) up to three weeks. In 25 patients (30.9%) they lasted during onset of stroke. We did not observe any case when such headache finished or abated before the stroke onset.

We compared several possible triggers and probable risk factors between patients with and without sentinel headache. Possible trigger factors for sentinel headache during 7 days before stroke are presented in Table 5 and factors which can be risk factors for sentinel headache - in Table 6. We found that attack of arrythmia during seven days before stroke (*p* = 0.04, OR 2.3; 95% CI 1.1–4.8) was the only trigger significantly associated with sentinel headache compare to patients without sentinel headache. The following probable risk factors were prevalent in patients with sentinel headache: female sex (*p* = 0.001, OR 2.4; 95% CI 1.5–2.9), atrial fibrillation (*p* = 0.04, OR1.9; 95% CI 1.05–3.3), angina pectoris (*p* = 0.01, OR 2.0; 95% CI 1.2–3.2), previous history of headache (*p* < 0.001, OR 4.0; 95% CI 2.0–8.0) including migraine and tension type headache.

**Table 5:** Possible trigger factors in patients with and without sentinel headache during 1 week before stroke

Possible trigger factors	Patients with sentinel headache before stroke (*n* = 81)	Patients without sentinel headache before stroke (*n* = 469)	Р, OR, 95% CI
Аlcohol consumption	15 (18.5%)	77 (16.4%)	0.6
Increased physical activity	17 (20.9%)	69 (14.7%)	0.2
Psychological stress	17 (20.9%)	112 (23.9%)	0.7
Overwork	17 (20.9%)	100 (21.3%)	0.9
Lack of sleep	12 (14.8%)	83 (17.7%)	0.6
Hypertonic crisis	48 (59.2%)	297 (63.3%)	0.6
Attack of arrhythmia	11 (13.6%)	30 (6.4%)	0.04 , 2.3; (1.1–4.8)
Overheating	4 (4.9%)	23 (4.9%)	0.8
Clinical infection	2 (2.5%)	27 (5.8%)	0.3

**Table 6:** Probable risk factors in patients with sentinel headache compare to the patients without sentinel headache during one year before stroke

Associated risk factors	Patients with sentinel headache before stroke (*n* = 81)	Patients without sentinel headache before stroke (*n* = 469)	Р, OR, 95% CI
Mean age	62,4	63,2	0.99
**Females, n (%)**	**52 (64.2%)**	**200 (42.6%)**	**0.001** **2.4; (1.5–2.9)**
Smokers, n (%)	25 (30.8%)	211 (44.9%)	0.02 0.6; (0.3–0.9)
Аlcohol consumption, n (%)	19 (23.4%)	149 (31.7%)	0.2
Low alcohol beverage, n (%)	11 (13.6%)	69 (14.7%)	0.9
Strong alcohol beverage, n (%)	12 (14.8%)	107 (22.8%)	0.1
Diabetes mellitus, n (%)	11 (13.6%)	73 (15.5%)	0.7
**Atrial fibrillation**, n (%)	**19 (23.4%)**	**66 (14.1%)**	**0.04** **1.9; (1.05–3.3)**
Body mass index > 25, n (%)	57 (70.4%)	316 (67.4%)	0.7
Low physical activity, n (%)	37 (45.7%)	170 (36.2%)	0.1
**Angina pectoris, n (%)**	**32 (39.5%)**	**117 (24.9%)**	**0.01** **2.0; (1.2–3.2)**
Myocardial infarction, n (%)	9 (11.1%)	44 (9.4%)	0.7
Arterial hypertension	77 (95.0%)	437 (93.2%)	0.7
Stroke in first degree relatives, n (%)	30 (37.0%)	177 (37.7%)	0.5
Peripheral artery disease, n (%)	3 (3.7%)	10 (2.1%)	0.6
Large-artery atherosclerosis, n (%)	18 (22.2%)	136 (28.9%)	0.2
**Previous history of headache**, n (%)	**71 (87.7%)**	**299 (63.8%)**	**< 0.001** **4.0; (2.0–8.0)**
Mean age of onset of previous headache	37.8	42.9	0.8
**Previous history migraine**, n (%)	**22 (27.2%)**	**62 (13.2%)**	**0.002** **2.5; (1.4–4.3)**
**Previous history of TTH**, n (%)	**63 (77.8%)**	**281 (59.9%)**	**0.003** **2.3; (1.3–4.1)**

## Discussion

The major findings of the present study were: 1) the prevalence of any headache during seven days before admission was significantly higher in stroke patients than in controls; 2) among these headaches a new type of headache and a previous headache with changes of characteristics represent sentinel headache; 3) migraine-like headache prevailed among new types of headache before stroke; 4) attack of arrythmia can be a trigger factor for sentinel headache before ischemic stroke; 5) significant factors associated with sentinel headache were: female sex, atrial fibrillation, angina pectoris, previous history of migraine and tension type of headache.

### Which headaches are a warning symptom of stroke (sentinel headache)?

Headache is very frequent in the general population. Approximately 95% of women and 91% of men have had at least one headache episode during the last 12 months. Therefore, a simple description of headache before stroke using stroke registry records without direct interview about previous headache as given by other authors [3] is not enough to show causality. A large simultaneous control group is necessary and only headaches that are significantly more prevalent in stroke patients than in controls can be called sentinel. To make the diagnosis of sentinel headache we subdivided headaches into a new type of headache never encountered before, a type of headache already encountered but with altered characteristics (migraine-like or tension type-like headache) and headaches previously encountered with no changes in characteristics. The two first groups were very rarely seen in the control group during seven days before examination (in comparison to seven days before stroke). Thus, a new type of headache and a headache with altered characteristics are true sentinel headaches causally related to stroke. Since they occur very rarely in the control group, they will also occur very rarely in the general population. The diagnosis of sentinel headache requires, however, that doctors take more care in eliciting the history of past headaches and compare them to a detailed history of the headache leading to the consultation. This is particularly relevant in emergency rooms where many patients present with a new or unusual headache.

### What are the possible mechanisms of sentinel headache before ischemic stroke?

The exact mechanism of sentinel headache is not established in ischemic stroke, but embolism seems most likely. We found an increased prevalence of attacks of arrythmia and of atrial fibrillation in patients with sentinel headache compared to patients without. Emboli impact on the vascular endothelium of cerebral arteries because they liberate cytokines which dilate the vessels and in addition are also proinflammatory. This might affect perivascular nerve endings and lead to nociception.

There is almost no literature to support the possible mechanism of headache in ischemic stroke. A case report described severe headache with an ipsilateral embolus in the proximal middle cerebral artery [6]. It was suggested that headache was caused by the proximal location of the embolus in the pain sensitive part of the artery. But such a mechanism cannot explain sentinel headache which is not associated with neurological deficit. Other authors have proposed dilatation of cerebral arteries caused by embolus or thrombus irritation of the pain sensitive arterial wall or by platelet release products, such as serotonin and prostaglandins which have been implicated in migraine pathogenesis [3].

Sentinel headache prevailed in women. Women are more prone to headache/migraine in general and to sentinel headache. It is also unsurprising that a previous primary headache increases the risk of sentinel headache. Some previous headaches might have been missed because patients with rare headaches tend to forget them.

### Clinical consequences of the present results

There is no established protocol for the handling suspected sentinel headache. We suggest the following algorithm: in a patient with an unusual, severe headache, it is necessary to compare all characteristics (intensity, duration, character, aggravation factors and accompanying symptoms as well as effect of drugs) with patient’s previous headache. If it is a new type of headache or a headache with altered characteristics in a person over 50 years of age, this should alarm about the possibility of sentinel headache and other serious pathology. These signs are so-called “red flags”. Such headache should make the doctor consider an immediate vascular investigational program. The intensity of the program must depend on the novelty and severity of the headache but as a minimum CT scan, ultrasonography of arteries on the neck and *electrocardiogram* should be performed. If possible, Holter monitor, MRI or MR/CT angiography of neck and cerebral vessels should be done.

### Strengths and weaknesses of the present study

This study included a big number of patients with first-ever stroke studied prospectively soon after the acute cerebrovascular event and a large simultaneously interviewed control group. It is the first study that conclusively demonstrates the existence of sentinel headache in ischemic stroke. It uses the international classification of headache disorders to describe both previous and current headaches and allows to give clinically important recommendations.

We tried to avoid investigator bias in our study by use of the same semi-structured interviews in both the stroke group and the control group and we did not mention a relation to stroke. As the stroke and control patients did not have major cognitive or language problems, it seems highly unlikely that recall bias would differ between the stroke group and the control group. The control group was admitted to the same emergency room and it was matched for age but not for sex. Males prevailed in the group of patients with stroke but females in the control group because they were more frequently admitted to the emergency room and we studied patients and controls in parallel.

The higher one-year prevalence of tension-type headache in controls (70%) than in stroke patients (62%, *p* = 0.06) may have several reasons. Stroke patients were more often males who generally have less headache. Furthermore, they had a brain disease and even if patients with obvious cognitive deficit were excluded, it seems likely that stroke patients less well may have remembered tension-type headaches which are not as severe as migraine. The seven days window was selected to better allow recall of the characteristics of headache. If a one-month window had been used there would probably have been more sentinel headaches but also more recall bias. On the other hand, most sentinel headaches were seen during the last 2–3 days before stroke. The study was controlled but, it was not blinded. That would have been ideal but not feasible for such a large study of acutely admitted patients.

The proportion of cardioembolic stroke was lower than expected. The main reason for this is absence of 48-h or 7 days monitoring of EKG since 24-h monitoring of the heart rhythm is too short to capture all. If it could be possible to make prolonged monitoring, the percentage of cardioembolic stroke could be higher. In such case the prevalence of atrial fibrillation as a possible risk factor and attacks of arrythmia as a trigger of sentinel headache could be even higher.

Patients were included consecutively at an emergency room, but it was necessary to exclude a majority of patients as described. Patients with impaired consciousness, cognitive and language impairment were excluded from the study because it was impossible to ascertain sentinel headache and previous history of headache in those patients. Some patients with left hemispheric stroke were excluded because of this reason. These are limitations of the generalizability of the study findings to the overall population of ischemic stroke.

## Conclusions

A new type of headache and a previous kind of headache with altered characteristics during one week before stroke are significantly more prevalent in stroke patients than in controls. These headaches represent sentinel headaches. Sudden onset of such headaches should alarm about stroke.

## Data Availability

The datasets used and analyzed during the current study are available from the corresponding author on reasonable request.

## Abbreviations

CT: Computed Tomography
MRI: Magnetic resonance imaging
DWI: Diffusion-weighted imaging
TIA: Transient ischemic attacks
SAH: Subarachnoid haemorrhage
OR: Odds ratio
CI: Confidence interval
mm Hg: Millimeters of mercury
ICHD-3: International Classification of Headache Disorders
TOAST: Trial of Org 10,172 in Acute Stroke Treatment
NIHSS: National Institutes of Health Stroke Scale
